# NESTML: a generic modeling language and code generation tool for the simulation of spiking neural networks with advanced plasticity rules

**DOI:** 10.3389/fninf.2025.1544143

**Published:** 2025-06-04

**Authors:** Charl Linssen, Pooja N. Babu, Jochen M. Eppler, Luca Koll, Bernhard Rumpe, Abigail Morrison

**Affiliations:** ^1^Simulation and Data Lab Neuroscience, Jülich Supercomputer Centre, Institute for Advanced Simulation, Jülich-Aachen Research Alliance, Forschungszentrum Jülich GmbH, Jülich, Germany; ^2^Institute for Advanced Simulation IAS-6, Forschungszentrum Jülich GmbH, Jülich, Germany; ^3^Department of Computer Science 3 - Software Engineering, RWTH Aachen University, Aachen, Germany

**Keywords:** model, neuron, synapse, simulation, spiking neural network, modeling

## Abstract

With increasing model complexity, models are typically re-used and evolved rather than starting from scratch. There is also a growing challenge in ensuring that these models can seamlessly work across various simulation backends and hardware platforms. This underscores the need to ensure that models are easily findable, accessible, interoperable, and reusable—adhering to the FAIR principles. NESTML addresses these requirements by providing a domain-specific language for describing neuron and synapse models that covers a wide range of neuroscientific use cases. The language is supported by a code generation toolchain that automatically generates low-level simulation code for a given target platform (for example, C++ code targeting NEST Simulator). Code generation allows an accessible and easy-to-use language syntax to be combined with good runtime simulation performance and scalability. With an intuitive and highly generic language, combined with the generation of efficient, optimized simulation code supporting large-scale simulations, it opens up neuronal network model development and simulation as a research tool to a much wider community. While originally developed in the context of NEST Simulator, NESTML has been extended to target other simulation platforms, such as the SpiNNaker neuromorphic hardware platform. The processing toolchain is written in Python and is lightweight and easily customizable, making it easy to add support for new simulation platforms.

## 1 Introduction

**Figure 1:**
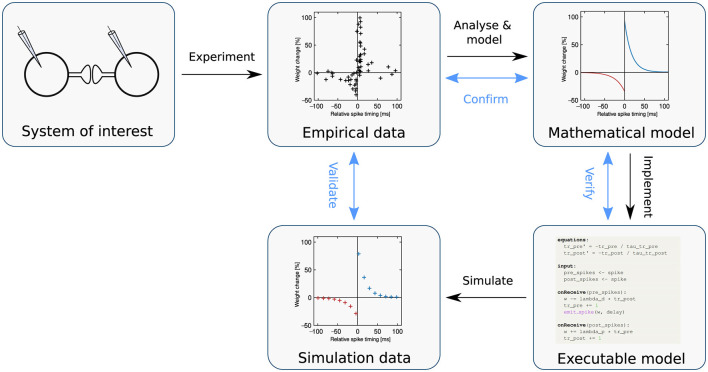
The modeling process. Following the black arrows, a biophysical system of interest is identified (**top left**), and through experiment, empirical data is generated (**top middle**). By analyzing and modeling this data, the system of interest is formulated as a mathematical model (**top right**). The model is then implemented as an executable computer simulation (**bottom right**). Simulation produces measurable quantities that are analogous to the original system (**bottom middle**). For example, the system of interest could involve a synapse; produce the data points (indicated as markers); be mathematically modeled according to Equations 9–11 and expressed as an executable model in NESTML (Listing 3), which when simulated, yields the data points on the **bottom middle**. Following the blue arrows in the diagram, *validation* is the process of evaluating the fit between simulated and empirical data, whereas *confirmation* is the process of checking that the mathematical model adequately captures the empirical data, and *verification* is the check that the executable model is an adequate implementation of the mathematical model. Note that these activities may be denoted differently across disciplines or even within computational neuroscience (see, e.g. Uhrmacher et al., 2024). Adapted from Trensch et al. (2018), their Figure 1B.

Numerical simulation is an essential technique for gaining insight into how neural network dynamics relate to brain function (Einevoll et al., 2019); it can also be deployed in an application-oriented way, for instance in machine learning or robotics tasks. To perform a simulation, an executable dynamical model is required: a representation of a natural process or phenomenon formulated precisely as an algorithm (Figure 1). Typically, this executable model is derived from a mathematical model developed to capture the salient properties of the system of interest, on the basis of empirical data.

In order to progress efficiently in science, all the processes identified in Figure 1 should be swift, accurate, and reproducible. As neural network models can be very large (up to hundreds of millions of neurons and three orders of magnitude more synapses; Jordan et al., 2018), much effort has been expended in recent decades in optimizing the *simulate* process in Figure 1.

Consequently, a wide range of neural network simulation software and hardware now exists. This benefits the field of computational neuroscience, as it provides researchers with the flexibility to choose a simulator that is best for addressing a particular research question (Crook et al., 2012). Although there is overlap in the types of models that each can simulate, each simulator typically provides its own unique user interface and strikes a different balance between efficiency, flexibility, and scalability. Many simulators offer the benefits of being well-characterized, using a diverse array of automated tests and benchmarks, being updated on a regular release cycle, and benefiting from the open-source model of iterative refinement (Zaytsev and Morrison, 2013). This diversity is advantageous, as any given tool will have flaws, such as software bugs, systematic biases, or unexamined assumptions, that may only become apparent in particular circumstances (Brette et al., 2007). Therefore, to increase the likelihood of long-term reproducible results, it is desirable that network models can be simulated using more than one simulator and the results cross-checked.

However, the fact that a wide diversity of simulation engines exists, complicates the exchange of computational models, both between labs and between simulators. Lack of standardized terminology, notation, and graphical representations for documenting models and networks negatively impacts progress in research (Nordlie et al., 2009; Pauli et al., 2018; Senk et al., 2022). Configuration files, scripting languages, or graphical interfaces used for specifying model structure can be very different for the different simulators, and this, together with subtle differences in the implementation of conceptually identical ideas, makes the conversion of a model from one simulation environment to another an extremely non-trivial task. Consequently, it is rarely undertaken, despite its obvious benefits and the sterling efforts of the ReScience initiative (Rougier et al., 2017). The field of computational neuroscience has much to gain from the ability to easily simulate a model with multiple simulators (Einevoll et al., 2019). For small-scale simulations, sometimes custom simulation engines are written. Unfortunately, these self-made frameworks, besides possibly duplicating published and established routines, are more likely to contain bugs and lack documentation, for instance, on edge-case behavior. Therefore, even for small networks, it is preferable to use standardized simulators and model description formats.

**Figure 2:**
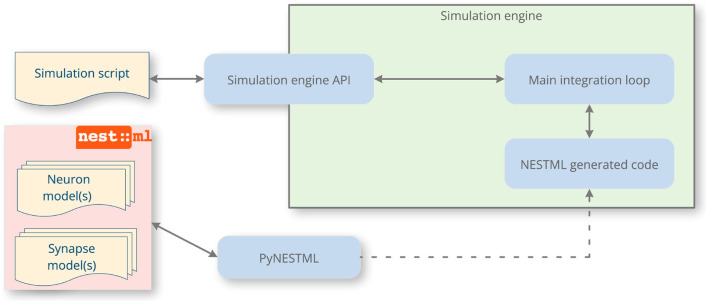
A NESTML-enhanced simulation workflow. Through code generation, NESTML augments the simulation engine with new neuron and synapse models. After writing the models, the end user invokes the PyNESTML toolchain, which generates and compiles code that will be executed by the target machine, and informs the user about any potential issues with the model via helpful messages. The simulation engine API is used to instantiate a network of the neurons and synapses defined by NESTML. When the simulation runs, the main integration loop of the simulator invokes the NESTML-generated code and returns recorded data back to the user via its API.

In this article, we address the issues of reproducibility and standardization in spiking neural network simulations by focusing on improving the speed and accuracy of the *implement* process in Figure 1. To this end, we present NESTML, a domain-specific language (DSL; Uhrmacher et al., 2024) for neurons and synapses (Plotnikov et al., 2016). It has a precisely defined syntax and semantics, captured in the formal grammar and extensive documentation, and further enforced by the toolchain through automated checks. NESTML encourages generality in the model descriptions by allowing end users to write models without having to consider implementation details and the simulation platform on which the model will ultimately be simulated. Likewise, the language encourages completeness in model descriptions, because each model contains all the necessary information needed for simulation. These properties of generality and completeness of the language benefit reproducibility. The language is designed with an emphasis on user-friendliness, being easy to read and write, while allowing powerful concepts to be expressed directly in the language syntax, such as ordinary differential equations, event handlers, and update statements in the style of imperative programming. It is strongly typed, incorporating physical units. Furthermore, the language is supported by a code generation toolchain that parses and verifies the correctness of the model and automatically generates simulation code for a given target platform (Figure 2); for example, C++ code targeting NEST Simulator (Gewaltig and Diesmann, 2007). Code generation allows an accessible and easy-to-use language syntax to be combined with good simulation performance; a technique that is increasingly gaining traction within neuroscience (Blundell et al., 2018a).

While these features of a DSL are convenient to have for neuron models, they are particularly useful in the case of complex synapse models, which are typically more challenging to implement than neuron models. Even a long-established model such as STDP (see Section 3.1.2) is tricky to implement correctly, especially on a distributed system, as it requires meticulous bookkeeping of spike times and communication latencies (Morrison et al., 2005). This only becomes more complex as further features such as additional postsynaptic variables or third-factor signals are introduced. As new variants of synaptic plasticity models are frequently introduced in the computational neuroscience literature, tools that support rapid development by abstracting away the tortuous algorithmic bookkeeping are highly desirable. Furthermore, some synaptic plasticity rules require special support from the neuron(s) that the synapse is connected to, such as additional buffers storing various trace values. Adding support to each neuron model for each synapse model is infeasible due to the number of possible combinations. Code generation allows the necessary data structures to be flexibly inserted into the neuron during code generation, allowing each neuron model to be combined with each synapse model without having to make changes by hand.

NESTML was first developed as a domain-specific language for neuron models for NEST simulator (Plotnikov et al., 2016). The NESTML toolchain itself was originally developed in Java based on the MontiCore Workbench (Krahn et al., 2010), which uses an extended version of the Extended Backus–Naur form (EBNF) to specify the grammar and automatically generates a parser for the model. The low adoption rate of Java in the neuroscience community made it difficult for researchers to maintain and extend the software. As a result, NESTML was re-implemented in Python (Perun et al., 2018; Blundell et al., 2018b), a widely used programming language in the neuroscience community. In the new implementation, the lexer and parser are generated using ANTLR (Parr, 2013), while still adhering to the software design principles of MontiCore.

Since the transition, the toolchain has become more modular and extensible. One advantage of this is that it has become much easier to support new simulation platforms, for example, the SpiNNaker neuromorphic platform (Furber et al., 2014; Linssen et al., 2023). A second advantage is that the scope and feature set of the toolchain could be rapidly expanded. Most notably, NESTML now provides the ability to define synapse models. The code for neuron and synapse models is generated in tandem, by automatically moving the relevant variables from synapse to neuron model (see Section 2.3). NESTML supports a number of synaptic plasticity rules such as spike-timing dependent plasticity (STDP), and variants thereof, such as STDP modulated by a third-factor such as a global dopamine concentration. Further feature enhancements include new language elements for spike event handling and generation, support for vector operations, faster code generation and build, and the addition of fundamental mathematical functions such as ceil and erf. There have been corresponding expansions and clarifications to the documentation of the toolchain, user guides, and teaching material in the form of tutorials (Linssen et al., 2024).

In the rest of this article, we first discuss other related modeling languages and code generation tools, then provide a detailed description of NESTML and its processing toolchain PyNESTML (Section 2), and then illustrate the usage and performance of NESTML for a specific, representative use case (Section 3).

### 1.1 Related work

NESTML fills a niche in the ecosystem of neural network modeling languages such as NeuroML/LEMS (Gleeson et al., 2010; Cannon et al., 2014), NineML (Raikov et al., 2011), and NMODL (Hines and Carnevale, 2019), which are predominantly used within the (computational) neuroscience domain, the Systems Biology Markup Language (SBML; Hucka et al., 2003), used for representing models of biochemical reaction networks, CellML (Miller et al., 2010) for models of cellular and subcellular processes involving interacting biomolecules, and Modelica (Modelica Association, 2023) for general modeling of physical systems. In general terms, DSLs can be *declarative*, which means they consist of a list of declarations corresponding to parts of the model, or, in contrast, they could consist of a list of *imperative* statements, giving a list of instructions for building the model in a stepwise manner.

NESTML is similar in conception to NMODL, as both freely allow the specification of imperative statements, encapsulated in a declarative model, combining the strengths of both model description approaches. In contrast to NMODL, NESTML has a more Python-like, modern and accessible syntax, which does not mix model definition and implementation details. For example, in NMODL, numerical solver selection is defined as part of the model itself, whereas in NESTML, the issues of *what* the dynamical equations are and *how* they are to be integrated are kept separate. The formulation in the NESTML syntax is a closer mapping of the formal, mathematical, concepts (see Section 2.4) and the integration sequence is more clearly defined (see Section 2.2), whereas the NMODL language is not conducive to formulating a series of statements that are executed once per timestep; statements may be executed twice or more under control of the NEURON solver. Furthermore, NESTML has additional features, such as the ability to assign different priorities to event handlers, and allow conditional statements to be formulated in a timestep-independent manner (see the onCondition block in Section 2.4). As NEURON is a compartmental neuron model simulator (that is, where a neuron is simulated in a morphologically detailed manner), NMODL contains syntactical features to expose compartment-related functionality, which is not part of the NESTML vocabulary. However, components of compartmental models, such as ion channels, could in principle already be formulated using NESTML.

NeuroML (Sinha et al., 2024) is a model description language used to describe models of neurons, synapses, and networks. NeuroML provides a high-level, nested (hierarchical) structure to models in a machine-readable (XML) format. NeuroML uses a purely declarative approach to specify models in different levels of detail: Level 1 focuses on the anatomical structure of neurons (MorphML), Level 2 builds upon Level 1 and specifies the electrophysiological properties of neurons such as ion channels and synaptic conductances (ChannelML), and Level 3 describes the network structure (NetworkML). The underlying dynamical behavior of the NeuroML components is defined using LEMS (Cannon et al., 2014), a general-purpose language for describing models and their simulations. In contrast, NESTML combines the declarative and imperative approaches, allowing imperative statements to be specified within the declarative model.

Both NeuroML and NESTML support the specification of a hybrid dynamical system. For NeuroML, this is achieved by LEMS language elements such as Dynamics, OnEvent and OnCondition; cognates of these elements are found in NESTML, namely the equations and the update block for continuous dynamics and onReceive and onCondition for discrete events (see Section 2.1). Additionally, NESTML also provides the modeler with the advantage of being able to define imperative statements in a Python-like programming language within the update, onCondition, and onReceive blocks. NeuroML is designed to be extensible using LEMS, which enables the definition of new model elements based on existing elements such as cells, networks, synapses, inputs, and channels. Models defined in NeuroML can be simulated using a LEMS simulation file executed through Python (pyLEMS) and Java (jLEMS) interfaces. Additionally, the LEMS simulation files can be translated to other simulation platforms including Brian2 (Stimberg et al., 2019), NEURON (Gleeson et al., 2010; Cannon et al., 2014) and EDEN (Panagiotou et al., 2022). Unlike NeuroML, NESTML has no language elements to support the specification of networks: it generates low-level code for individual neuron and synapse models. These individual components can then be further instantiated to build a network and simulate on the target simulator with the help of a corresponding simulation script of the appropriate sort for the targeted platform (e.g PyNEST; Eppler et al., 2009).

As with Brian2, NMODL, and LEMS, all definitions and expressions are strongly typed in NESTML. The toolchain checks the consistency of physical units during model parsing and validation, which prevents the use of inconsistent expressions. NESTML automatically adds scaling factors during code generation when the definitions have consistent units (see Section 2.4.1).

NESTML consists of a combination of modeling language and code generation toolchain, providing the advantage of developing both in parallel. The category of code generation tools in neuroscience also includes Brian2 (Stimberg et al., 2019), GeNN (Yavuz et al., 2016), NeuroML (Sinha et al., 2024), and NMODL (Kumbhar et al., 2020; Abi Akar et al., 2019). Each of these has its own separate toolchain and APIs and targets different simulation platforms. All the mentioned code generation tools primarily focus on code generation for their respective simulation platforms. Notably, Brian2GeNN (Stimberg et al., 2020), an interface that combines the code generation capabilities of Brian2 and GeNN, bridges the capabilities of the two simulators, enabling Brian2 models to be executed on GPUs. NESTML takes a more simulator-agnostic approach, generating code that can be run on multiple backends such as NEST (Gewaltig and Diesmann, 2007) for CPU-based simulations, and the SpiNNaker neuromorphic platform (Furber et al., 2014). Furthermore, the NESTML toolchain is modular and can be easily extended to add support for other simulation platforms (see Section 2.5.4). Currently, support for a GPU backend, NEST GPU (Golosio et al., 2021), is under development. This extensibility is made possible by NESTML's clear separation between model specification and its subsequent instantiation and simulation in a network.

Note that in the terminology of object-oriented programming, NESTML formalizes the *classes*, but not the *instances* of each class, which can vary in their state, parameter values, and connectivity, but not (beyond changing parameter values) their behavioral repertoire. Instantiating the actual populations and connecting the elements together in a network at runtime remains the responsibility of the simulation engine for which NESTML generates code. For this, an imperative specification approach such as PyNN (Davison et al., 2009) or PyNEST (Eppler et al., 2009) can be used.

Furthermore, NESTML can be used in a complementary fashion to other standards, such as the Simulation Experiment Description Markup Language (Köhn and Le Novère, 2008), a DSL that formalizes the simulation runs (duration, iterations, parameter sweeps, etc.) and standards for uniquely identifying components and processes, such as the Systems Biology Ontology and the Computational Neuroscience Ontology (Whetzel et al., 2011).

## 2 Methods

### 2.1 Mathematical models of hybrid systems

Essentially, NESTML is a modeling language for hybrid dynamical systems. Hybrid systems may contain a set of discrete variables that take on a number of discrete states, and continuous-valued variables (typically, real-valued numbers) that are allowed to “jump” (change) instantaneously as the result of an event (Shampine and Thompson, 2000). An event has a precise timestamp of occurrence, but no duration or dynamics in time. This approximation allows the efficient communication of event occurrences rather than the more computationally expensive alternative of communicating real-valued data at each simulation timestep. In the specific case of spiking neural networks, neuronal action potentials are often approximated as events and can be conveniently expressed mathematically by the use of a Dirac delta function δ(*t*), so that the spike train of a neuron can be written as:


(1) $$\begin{array}{l} s\left(t\right)=\underset{k}{\sum }\delta \left(t-t_{k}\right) \end{array}$$

where *t*_*k*_ is the *k*-th spike of a given neuron. When spikes are treated as events, neuron and synapse models are therefore in the class of hybrid dynamical systems defined above. The continuous-time dynamics are expressed as a system of ODEs, that govern the dynamics of a vector of state variables **x**, while receiving an arbitrary external input **g**(*t*):


(2) $$\begin{array}{l} \frac{\text{d}\text{x}}{\text{d}t}=\text{f}\left(t,\text{x}\right)+\text{g}\left(t\right) \end{array}$$

Handling and generating the discrete-time events can be expressed either as a convolution of the events with a kernel inside the ODEs, or as a set of conditions and actions. Often these two approaches are equivalent and the choice of how to express them is largely a matter of personal preference. For example, an exponential postsynaptic current can be calculated using two approaches: either by convolving a decaying exponential kernel (given as a function of time) with the incoming spike train, or by modeling the current as an ODE that undergoes exponential decay, with the corresponding state variable incremented upon the arrival of a spike by an event handler. In the latter case, a condition/action block can be used:


(3) $$\begin{array}{l} \text{if}\;condition\;\text{then}\\ \;statements\\ \text{endif} \end{array}$$

Besides receiving a spike, conditions can include propositional logic on the values of state variables, and can also be triggered by an advancing of the simulation by a fixed timestep. Statements typically modify state variables, using standard mathematical operators and functions, and can also involve loops and recursion (a list of the types of statements that can be used is given in Supplementary Table 1).

### 2.2 Numerical integration of models in time

**Figure 3:**
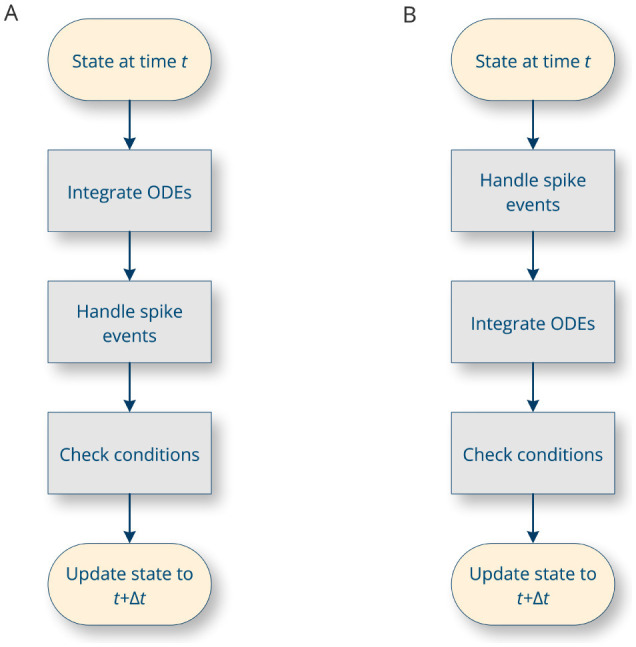
The main integration loop in a spiking neural network simulator. The precise sequence of operations carried out depends on whether the simulation is considered to have propagation delays **(A)** or not **(B)** (Morrison and Diesmann, 2008).

The general strategy for integrating a hybrid system numerically is to first integrate the ODE until the time of the next event, and then incorporate new events (Morrison and Diesmann, 2008). In between, conditions are evaluated, such as whether the membrane potential has exceeded the spike threshold, and the corresponding statements are executed. These steps are carried out in a loop by the simulation platform. Depending on the specifics of the platform, there are several possible approaches. In particular, the presence or absence of transmission delays in the system affects the order of processing steps (Figure 3A vs. Figure 3B). In addition, the loop can proceed in timesteps of constant duration (time-driven simulation, in which case Δ*t* is a constant value), or it can jump directly from event to event (event-driven simulation, in which case Δ*t* is the time between two successive events).

**Figure 4:**
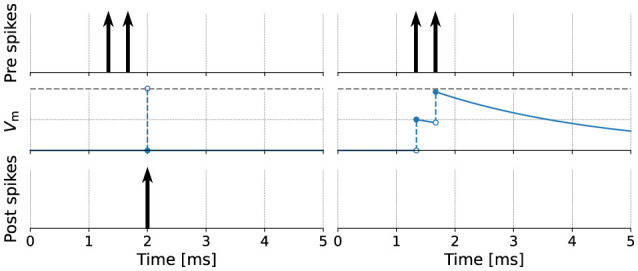
Numerical integration of an integrate-and-fire neuron. **(left)** Time-based simulation with resolution Δ*t* = 1 ms; spikes are processed at the next simulation timestep. The membrane potential threshold (dashed horizontal line) is crossed and a spike is emitted. **(right)** Event-based simulation of the same neuron; spikes are processed immediately as they arrive. No spike is emitted. Open and filled circles indicate open and closed intervals.

For time-driven algorithms, the choice of timestep is a trade-off between the numerical precision achieved (and possibly, numerical stability), and the time and computational effort required for the simulation. In spiking neural network simulations, forcing spikes to a fixed grid can result in discrepancies in the number of spikes a neuron produces. As an example, consider the integration of a dynamical system representing a simple integrate-and-fire neuron, where incoming spikes directly increase the membrane potential *V*_m_ (Figure 4). The left panels show a fixed timestep simulation, where spikes can only be processed at multiples of the simulation resolution. A coarse resolution of 1 ms was chosen to illustrate the effect. If a spike occurs inside the interval between two subsequent steps, its time of occurrence is effectively rounded up to the start of the next step. Because this rounding causes the spikes to be processed simultaneously, the threshold is crossed which causes a spike to be emitted by the postsynaptic neuron.

An event-driven approach allows spike times to be handled at machine precision, rather than being constrained to the simulation resolution, and should therefore be considered as an alternative simulation strategy for hybrid systems. In the panels on the right, spikes are processed immediately, and the intermediate exponential decay in *V*_m_ causes the neuron to not reach the threshold, so no spike is emitted. The downside of the event-driven approach is a lack of flexibility in the domain of spiking neural networks, as the dynamics of most neuron models are not invertible; thus the time of occurrence of the next spike cannot be analytically calculated, which may cause some threshold crossings to be not detected. Moreover, the overhead of maintaining a sorted event queue becomes substantial as networks increase in size (Lytton and Hines, 2005; Migliore et al., 2006). Hybrid strategies that are time-driven in nature, but still allow events to occur between timesteps, provide a potential solution. For example, to find the “exact” time of threshold crossing, a linear or higher-order interpolation can be made of the membrane potential between grid points, search algorithms such as binary search can be used (Morrison et al., 2007; Hanuschkin et al., 2010), and for some models, algebraic methods can be used (Krishnan et al., 2018). All of these approaches aim to approximate the basic integration scheme outlined before: in a loop, integrate continuous dynamics between events, and then handle the events, all while a set of conditions are continuously checked.

As different simulation environments may employ different integration strategies (Figure 3), and the choice of strategy is beyond the control of NESTML (as it depends on the specifics of the simulation platform), it is crucial for NESTML, as a generic modeling language, to maintain compatibility with each of these approaches. Models in NESTML are conceptually equivalent to “pure” hybrid, event-based systems (Section 2.1), allowing the most precise simulation strategies while retaining an open-ended compatibility. However, this implies that the same NESTML model can yield different simulation outcomes, depending on the choice of platform and its specific parameters such as the timestep resolution. Some common numerical issues associated with hybrid systems, such as numerical divergence, and issues related to zero crossing detection should be addressed by changing the integrator used and integration options inside the generated code (at compile time) or by setting the relevant options inside the simulation platform (at runtime). Furthermore, the ODE-toolbox can test the stiffness of a system of ODEs solved by a numerical integrator (see Section 2.5.3).

### 2.3 Interdependence of models

Synaptic and neuronal models should ideally be formulated independently of each other, so that each neuron can be combined with each synapse for maximum flexibility. When a synaptic plasticity rule is formulated as a computational model, the plasticity rule is often expressed as a function of the timing of pre- and postsynaptic spikes, which are used in the dynamics of the weight update for that particular rule (see Section 3.1.2 for an example). Note that as each neuron is typically connected to hundreds or thousands of other neurons via synapses on its dendritic arbor, each of those synapses will observe the same postsynaptic spike times, and store and numerically integrate them in exactly the same way, causing a very large redundancy in memory and computation.

To prevent this redundancy, these values should only be stored and computed once; ideally in the instances of the neuron models, where the spike timings are readily available. To achieve this, NESTML has the capability to process a synapse model as a pair together with the (postsynaptic) neuron model that it will connect to when the network is instantiated in the simulation. A list of these (neuron, synapse) pairs can be provided as code generator options (see Section 2.5.4) when invoking the NESTML toolchain to generate code. During code generation, state variables that depend only on postsynaptic spike timing are then automatically identified and moved from the NESTML synapse model into the neuron model by the toolchain. In the generated code, at the points where the respective variables are used by the synapse (for instance, where they are used in calculating the change in synaptic strength), the variable references are replaced by function calls into the postsynaptic neuron instance. All parameters that are only used by these postsynaptic dynamics (for instance, time constants) are also moved to reduce the memory requirements for the synapse. Detecting and moving the state, parameters, and dynamics (ODEs) from synapse to neuron is carried out fully autonomously. We refer to this feature as the “co-generation” of neuron and synapse. It enables flexibility and separation of concerns in the model formalisations without compromising on performance. Co-generation is further illustrated with a usage example in Section 3.1.

### 2.4 The NESTML language

Many of the principles of NESTML can be implemented irrespective of the precise underlying syntax used, which could have been based, for instance, on XML^1^ or YAML.^2^ The chosen NESTML syntax was inspired by Python and is designed to be minimalistic, simple and intuitive. For example, by not using XML tags, a large amount of syntactical overhead is avoided. NESTML is written in plain text, allowing users to edit models directly in any text editor. The language elements closely resemble mathematical models of neurons and synapses, enabling users to input ordinary differential equations (ODEs) directly as they are defined in the mathematical model (see Section 3.1 for example models).

The different elements of the language correspond to specific parts of the integration loop for hybrid dynamical systems (Figure 3). In general, models are hierarchically structured in elements or blocks. The top-level block names the model and can contain any of the following sub-blocks (an example neuron model with all the sub-blocks can be found in Listing 2).

- state: contains a list of declarations of variables with initial values that are updated as the simulation evolves. These can be variables with the dynamical equations in the equations block or variables with discrete-time dynamics (for example, finite state machines or Markov chains) that are updated over time in the update block.
- parameters: contains a list of parameter declarations. Parameters remain constant during the simulation.
- internals: contains a list of internal parameter declarations. Internals remain constant, just like parameters, but are not directly specified by the user; instead, they are derived from other parameters in the parameters block.
- equations: contains a list of differential equation definitions. Equations can be given as functions of time, first-order, or higher-order differential equations; the toolchain will attempt to rewrite the dynamics into a system of first-order differential equations, suitable for numerical integration. Additionally, the block may contain inline expressions to reduce redundancy and improve the legibility of the model code. Such an expression will be replaced verbatim when its variable symbol is used in subsequent ODEs. There is also support for delay differential equations, for which the necessary buffers are automatically generated.
- input: contains a list of input ports, each receiving either spike events or values that are continuous in time.
- output: defines the type of output this model generates, if any; for instance, the model can emit spikes.
- update: contains statements that are executed *between* events, corresponding to the “free-flight” integration of the system of differential equations. Depending on the simulation strategy, statements in this block are executed once every timestep, at a fixed, discrete simulation resolution (Figure 4, left) or once for every event, jumping from event to event (Figure 4, right). If there are ODEs that need to be integrated in time, statements in this block are responsible for performing the integration by calling integrate_odes(); a specific subset of ODEs can be integrated by passing the variable names as parameters. At the start of the block, the state corresponds to that at time *t*. At the end of the block, the state should have been updated (by the statements) to *t*+Δ*t*.
- onReceive: contains statements that are executed whenever an incoming spike event arrives; can be defined for each spiking input port. Optional event parameters, such as the weight, can be accessed by referencing the input port name. Priorities can optionally be defined for each onReceive block; these resolve ambiguity in the model specification of which event handler should be called after which, in case multiple events occur at the exact same moment in time on several input ports, triggering multiple event handlers.
- onCondition: contains statements that are executed when a particular condition holds. The condition is expressed as a (boolean-typed) expression. Having an explicit onCondition block, rather than writing conditions as part of the update block statements, means that conditions can be checked at various points in the integration loop. For example, referring to Figure 3B, the ODEs can be integrated for the full timestep length Δ*t*, after which conditions are checked; however, many numerical solvers take smaller, intermediate timesteps, and the conditions could in principle also be checked after each intermediate step.
- function: defines a helper function that takes arguments and contains statements, and returns a value through the return keyword.

The language elements of NESTML that can appear in each block are summarized in the Supplementary Table 1. Mathematical and logic operators are available to form complex expressions. Conditionals, loop statements and function calls can be used in writing imperative code. Several predefined functions are available, such as functions for random variables random_normal, random_uniform, and random_poisson, as well as mathematical functions like exp, ln, sin, cos, etc. Predefined variables such as *t*, which represents the global simulator time, and *e* which represents Euler's constant are also available.

#### 2.4.1 Physical units

If numerical models are developed in a general-purpose scientific computation environment such as Python, it is easily possible to accidentally define a model that does not make physical sense due to mismatches in the physical units in an expression (one recent case is described in Oberländer et al., 2022). To address this issue, all of the definitions and expressions in NESTML are strongly typed, which means they have the type integer (a natural number), real (a real number), string, boolean (a Boolean value), or a physical unit such as mV for millivolt or nS for nanosiemens. The types of expressions and assignments are checked for consistency when the toolchain is invoked, preventing users from writing models that are not internally consistent (see Listing 1 for an example). NESTML automatically adds a scaling factor of 10^−3^ when generating code for line 4 of Listing 1, because the units are consistent but the prefixes are different (millivolt and volt). NESTML also checks for consistency in expressions (lines 10–11) and raises an error when the units are incompatible (line 11). Internally, we use the AstroPy package for units computations (Astropy Collaboration et al., 2022). These are supplemented with a set of equivalences, such that any quantity is convertible to real, and that real numbers are convertible to integers (although a warning will then be emitted during model validation).

**Listing 1:**
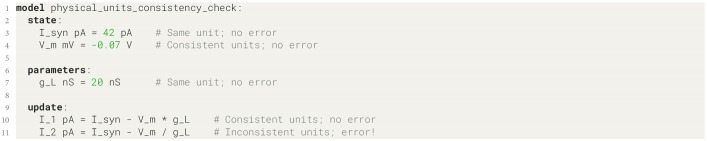
Demonstration of physical units consistency check.

All input spikes are modeled as Dirac delta functions in time, with an implicit unit of s^−1^. Physical units such as mV and nS can be directly multiplied by the Dirac delta function to model an input spike with a physical quantity such as voltage or conductance.

#### 2.4.2 Vectors

NESTML provides support for declaring variables as vectors to store an array of values. They can be defined in the parameters, state, and internals blocks. The vectors are declared with a non-zero size and can be any of the NESTML types or physical units described in the previous section. The size of a vector can be a fixed, positive integer value, or a variable previously declared in the parameters or internals block. In this case, the vector will be resized if the value of the size variable changes during the simulation. The vector variables are particularly beneficial when the model has to capture and perform computations based on a sequence of values. For example, Hagen et al. (2022) implemented a neuron model using NESTML which computes the finite impulse response (FIR) filter of incoming neuronal spikes. The model stores FIR filter coefficients and binned input spikes into vector variables and calculates the filter output at every timestep of the simulation.

#### 2.4.3 Comments and docstrings

Single or multi-line comments in the model are supported with the # character. Comments following a declaration on the same line are considered to document the variable that was declared. In combination with *docstrings*, which are reStructuredText-formatted, human-written, and human-readable model documentation strings (Goodger and van Rossum, 2001), these allow us to produce richly formatted model documentation pages in HTML in a fully automated manner.^3^

### 2.5 The toolchain PyNESTML

**Figure 5:**
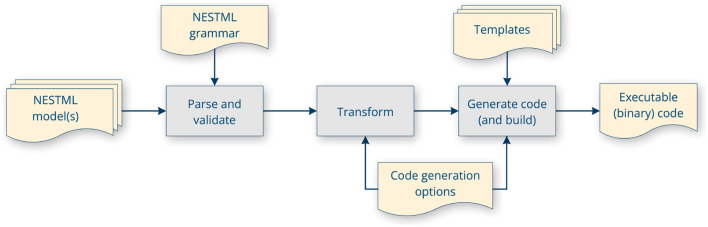
PyNESTML toolchain internal workflow: parsing, validation, transformation, code generation using templates and code generator options, and optionally, building the code.

The PyNESTML toolchain is illustrated in Figure 5 and consists of several sequential processing steps, as described in detail below, which result in code being generated and then built. The output of the toolchain depends on some invocation parameters. These include, most importantly, which target platform to generate code for, where the generated code should be stored and (optionally) installed to, logging and verbosity options, and options specific for each target platform code generator.

#### 2.5.1 Parsing

The NESTML model is parsed to an internal representation, called the parse tree, using a lexer and parser. The lexer takes in the model file and generates a set of tokens including keywords, operators, and string literals. The parser takes in the tokens and generates a parse tree based on the grammar rules for all the constructs used in the NESTML language. The NESTML grammar is expressed as an Extended Backus-Naur form (EBNF) grammar (Parr, 2013); we use ANTLR (ibid.) to automatically generate the Python code for the lexer and parser, which in turn will lex and parse the NESTML model files.

A parse tree represents an elementary approach for storing models in a convenient computer-processable structure. However, often additional information or operations need to be stored together with the nodes in the tree, making the immutable structure of the parse tree not useful for further processing. Thus, an intermediate representation of the model is derived called the Abstract Syntax Tree (AST; Hölldobler et al., 2021). While a given node in the parse tree is represented by a token object as generated by ANTLR, a node in the AST is a data structure that stores the information and operations for individual elements of the NESTML language. All the nodes in the AST inherit from an abstract base class called ASTNode. These node classes are hand-written to enable custom functionality (methods and members) in different types of nodes, but in general they are lightweight and can be easily adapted from existing node classes.

NESTML makes use of the *visitor* design pattern. Each visitor is a separate class that operates recursively on nodes in an AST. It is a useful software architecture pattern that separates the concern of operating on the AST from that of representing it. New operations can be easily added by defining new visitors without changing the existing code of the tree nodes. Each visitor class has a single responsibility and implements a specific operation, making the code more maintainable and understandable. The visitor pattern is used for validation (Section 2.5.2), transformation (Section 2.5.3), and code generation (Section 2.5.4).

Because NESTML supports scoped declarations, where a *local variable* is only accessible inside the block it is defined in, each block is endowed with a unique symbol table, and each node is assigned a particular symbol table scope. This pattern facilitates easy symbol lookup (Hölldobler et al., 2021).

#### 2.5.2 Validation

Validation consists of several steps that are run in sequence:

1. Parsing (validating the model syntax according to lexer and grammar). Validation of correctness is carried out by the parser itself, which is generated based on the NESTML grammar.
2. Further syntactical, but context-dependent (as opposed to the grammar's context-free) checks, for example, that names are uniquely defined within each scope.
3. Semantic checks, for example, consistency of physical units.
4. Checks during code generation (some target platforms may have specific requirements, such as a particular variable having been defined).

The second and third steps involve a set of checks, which often use the visitor pattern and test a model for correctness in one specific respect. Each of these checks is, like unit tests, hand-written by the NESTML developers. They are run immediately following the parsing of each model into an AST, and cover issues that might be legal according to the NESTML syntax, but semantically incorrect. A subset of these checks is run between model transformation steps, to help validate the correctness of the transformation (see Section 2.5.3). A list of the defined checks is given in the Supplementary Table 2.

#### 2.5.3 Transformation

Transformations operate on the AST of each model and can add, remove, or alter nodes in the tree. Transformers change the internal structure of the models to improve certain characteristics (such as runtime performance) without necessarily changing their observable behavior (input-output function; Mens and Gorp, 2006). Transformations can also refine the model specification into a more fully-fledged implementation, by means of successive refinement steps that add more concrete details. After each transformation stage, the altered ASTs can be printed again as NESTML syntax. This enables the model developer to inspect, for instance, how the solution of the ODEs has been implemented.

Transformers can be specific to model type (for instance, some optimizations could make sense for synapse models but not neurons). Analogously, a specific set of transformers (or a set of transformers parameterized in a certain way) can be required for specific target platforms. For instance, if the generated code is to be in C++, a “variable names transformer” converts variable names that would collide with C++ language keywords (but would be fine if we were generating code for, say, Python).

The call signature of a transformer is that it accepts a set of NESTML model ASTs (potentially, a mix of neuron and synapse models), and again returns a set of models. By allowing transformers to work on sets of models rather than individual models, components of each model can be processed together, enabling optimizations that would not be possible if each model were processed separately. For example, the neuron/synapse “co-generation” transformer (see Section 2.3) can move variables from a synapse to a paired neuron model.

**Figure 6:**
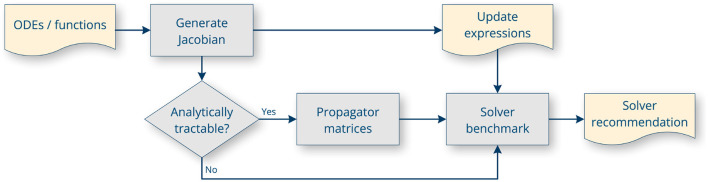
ODE-toolbox flow diagram. All inputs to and outputs from the package are encoded as strings in JSON files.

Transformations related to kernels and ODEs are carried out by the Python package ODE-toolbox (Linssen et al., 2022; Figure 6). It was spun off from NESTML as an independent Python package (Blundell et al., 2018b), but remains an essential dependency of the PyNESTML toolchain. It leverages SymPy (Meurer et al., 2017) for the symbolic manipulation of differential equations. For all dynamics admitting an analytic solution, ODE-toolbox generates propagator matrices that allow the solution to be calculated at machine precision (Rotter and Diesmann, 1999). For all other systems, first-order update expressions are returned based on the Jacobian matrix. ODE-toolbox can also perform solver benchmarking based on a set of user-configurable heuristics, to predict which solver will perform better during simulation.

#### 2.5.4 Code generation

Options specific to the code generator for a specific target platform are given as code generator options encoded in a JSON data structure, rather than being part of the model description itself. In general, we try to separate implementation details from the pure specification of the model. Implementation details could include the choices for timestep, numerical solver, random number generation algorithm, threshold crossing interpolation algorithm (if applicable), the integration sequence (Figure 3), or many other specifics. These details should not be part of the model specification itself, but are properties of the simulation platform, and should therefore only be incorporated during code generation (or even after that; such as a timestep that is set in the simulation script). Note that in principle one *could* write, for example, a root-finding algorithm in NESTML to find the exact time of threshold voltage crossing; this would then be considered essential for the model and a part of the specification rather than the implementation. Another example would be the support for electrical synapses (or gap junctions). For instance, NEST has a native implementation for electrical synapses; NESTML supports code generation for this feature through code generation options. In general, we recommend taking full advantage of the automated processing capabilities of the toolchain; specifying these details in the model description should be considered an action of last resort.

Support for a new target platform is as simple as adding a new set of templates, if *pretty printers* (Hölldobler et al., 2021) or *unparsers* (which convert nodes in the AST into target language code) for the target language are already available, as is the case for C, C++, and Python. Otherwise, new printers would have to be written, however, these can be easily adapted from the existing printers. NESTML, with the help of the ODE toolbox, generates the solutions for the ODEs in the model and recommends the type of solver for the given system of ODEs. The NESTML model is agnostic of the type of solver to use and relies on the implementation of a particular solver simulation platform. If the system of ODEs is analytically solvable with the help of propagators, NESTML recommends that solution and generates the relevant code to compute the state updates. On the other hand, if the system of ODEs is non-linear, the toolchain recommends a numerical solution, in which case, the implementation of a particular type of solver (for instance, forward Euler or Runge-Kutta) must be solely provided by the hosting simulation platform.

#### 2.5.5 Build

The build stage involves invoking a compiler and linker on the generated code, optionally incorporating libraries like the C++ standard library, or platform-specific libraries like those for numerical ODE solvers. Subsequently, any required installation steps are performed, such as copying files, running make install, or uploading the generated binary file(s) to a neuromorphic hardware platform. Logs from the build process can optionally be captured to stdout and stderr, but it should not be necessary to inspect these under normal circumstances; any potential issues with the model should have been detected and reported in a user-friendly manner during the parsing and validation step.

If the target platform has a means for dynamically loading the generated binaries at runtime, the models can be immediately loaded and instantiated in a network after code generation is complete (in NEST, this is realized by the nest.Install() API call). The NESTML model code can then even be included inside the simulation script itself. Otherwise, the simulation engine for the specified target platform will require recompilation as well, with the generated NESTML code statically linked.

## 3 Results

### 3.1 Neuron and synapse co-generation

To illustrate the use of NESTML, we present a use case where a specific neuron type and a specific synapse type are modeled and simulated. The neuron is an adaptive exponential integrate-and-fire type with spike-rate adaptation (Section 3.1.1), and the synapse is a spike-timing dependent plasticity rule (Section 3.1.2). Code for these models is generated in combination, demonstrating the “co-generation” transformer detailed in Section 2.5.3.

#### 3.1.1 Neuron model

The subthreshold dynamics of the neuron model is given by a set of two coupled differential equations, the first describing the dynamics of the membrane potential *V*_m_, and the second describing the dynamics of an adaptation current *I*_adap_:


(4a) $$\begin{array}{ll} \frac{\text{d}V_{\text{m}}}{\text{d}t} & =\frac{1}{C_{\text{m}}}\left(-g_{\text{L}}\left(V_{\text{m}}-E_{\text{L}}\right)+I_{\text{spike}}-I_{\text{syn}}-I_{\text{adap}}+I_{\text{stim}}\right) \end{array}$$

(4b) $$\begin{array}{ll} \frac{\text{d}I_{\text{adap}}}{\text{d}t} & =\frac{1}{\tau_{\text{adap}}}\left(a\left(V_{\text{m}}-E_{\text{L}}\right)-I_{\text{adap}}\right) \end{array}$$

For a complete listing of all parameters and their values, refer to Supplementary Table 3.

The spike current term *I*_spike_ is given by the expression:


(5) $$\begin{array}{ll} I_{\text{spike}} & =g_{L}\Delta_{T}\exp\left(\left(V_{\text{m}}-V_{\text{th}}\right)/\Delta_{T}\right) \end{array}$$

The synaptic input current is given by the convolution:


(6) $$\begin{array}{ll} I_{\text{syn}} & =\underset{i}{\sum }w_{i}\left(K_{\text{syn}}*s_{i}\left(t\right)\right) \end{array}$$

with *i* summing all presynaptic neurons, *w*_*i*_ weight of the connection from presynaptic neuron *i*, *s*_*i*_ the spike train emitted by neuron *i*, and the alpha-shaped (rise-and-decay) postsynaptic current kernel defined as (Rotter and Diesmann, 1999):


(7) $$\begin{array}{ll} K_{\text{syn}} & =\{\begin{array}{ll} (e/\tau_{\text{syn}})t\exp(-t/\tau_{\text{syn}})\; & t\geq 0\\ 0\; & \;\text{otherwise} \end{array} \end{array}$$

and *s*_*i*_(*t*) is the incoming spike train of presynaptic neuron *i*, defined as a sum of Dirac delta functions (see Equation 1), weighted by *w*_*i*_ (in units of Ampère). Alternatively, the spikes can be also directly integrated into the synaptic current (see the code listing in the Supplementary material, Listing 1, Line 12).

The differential equations expressing the subthreshold dynamics of the neuron are complemented by the membrane potential threshold condition for spike generation:


(8) $$\begin{array}{l} \text{if }V_{\text{m}}\geq V_{\text{peak}}\to \{\begin{array}{l} \text{a spike is emitted;}\\ V_{\text{m}}\text{ is set to }V_{\text{reset}}\text{;}\\ I_{\text{adap}}\text{ is incremented by }b. \end{array} \end{array}$$

Note that we consider the postsynaptic response to be, conceptually, a part of the neuron model. This is in line with current approaches in neuroscience simulators. Because the dynamics of the postsynaptic response is linear, the postsynaptic current contributions of all the synapses can be added into one single postsynaptic variable *I*_syn_ and integrated as one.

The model can be expressed in NESTML syntax as shown in Listing 2. Note the direct correspondence between the theoretical (mathematical) model and the model syntax, in particular between the ODEs and convolution with a kernel (Equations 4a–7) and the equations block, lines 6–12, as well as the event conditions (Equation 8) and the onCondition block on lines 36–39. Additional support is added for a continuous-time current input using the continuous input port on line 28.

**Listing 2:**
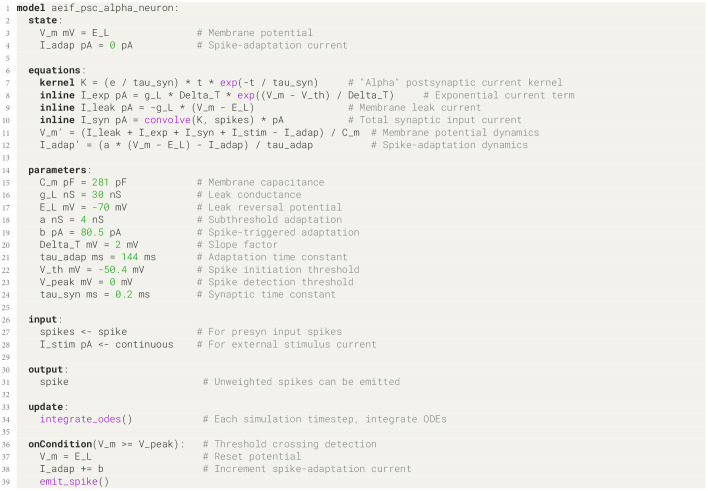
Adaptive exponential integrate-and-fire neuron model with alpha-shaped postsynaptic currents.

In this particular model, the dynamics of the postsynaptic response (*I*_syn_) will be integrated numerically for the sake of making comparisons in performance to NEST built-in models in benchmarks (Section 3.2), but an analytic solution for the alpha kernel dynamics is readily derived by ODE-toolbox; analytic solver code can, in general, be generated for any subset of ODEs that admit an analytic solution and is combined seamlessly with the numerical integration code. The variable that keeps track of the state of convolutions (here, I_syn) is automatically added to the set of state variables during code generation. Alternatively, the convolutions can be written using an onReceive event handler containing statements to process the spike input. The two methods of handling spikes are equivalent and the user can choose either to implement their models. For the sake of demonstrating the capabilities of NESTML, we show the convolutional approach in the neuron model (Listing 2) and the event handler approach in the synapse model (Listing 3). The alternative version of the neuron model using the event handler can be found in the Supplementary material (Listing 1).

**Listing 3:**
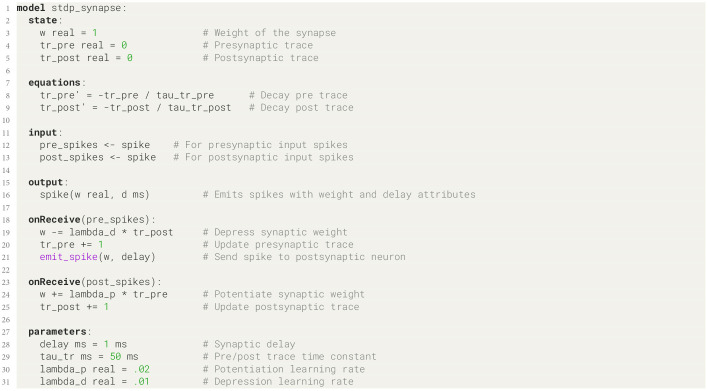
STDP synapse model.

#### 3.1.2 Synapse model

The synapse model that we present here is a variant of a spike-timing dependent plasticity (STDP) rule (Bi and Poo, 1998) and defined such that a pair of spikes in the pre- and postsynaptic cells, at times *t*_pre_ and *t*_post_ respectively, induces a change Δ*w* in the weight *w* of the synapse:


(9) $$\begin{array}{l} \Delta w=\;\lambda_{\text{p}}\cdot K_{\text{p}}\left(t_{\text{post}}-t_{\text{pre}}\right)\\ \;-\lambda_{\text{d}}\cdot K_{\text{d}}\left(t_{\text{pre}}-t_{\text{post}}\right) \end{array}$$

The weight is increased through the first term when *t*_post_>*t*_pre_ and decreased by the second term when *t*_pre_>*t*_post_. Coefficients λ_p_ and λ_d_ (≥0) set the magnitude of the update. The temporal dependence is defined by the filter kernels *K*_d_ and *K*_p_, which are taken to be decaying exponential functions:


(10) $$\begin{array}{l} K_{\text{p,d}}(t)=\{\begin{array}{ll} \exp(-t/\tau_{\text{p,d}})\; & t\geq 0\\ 0 & \text{otherwise} \end{array} \end{array}$$

To implement the behavior for the kernel, we use two extra state variables, which represent a pre- and postsynaptic “trace” that keeps track of recent spiking activity. These trace variables are incremented by 1 whenever a spike is generated and decay back to zero exponentially. Expressed as a differential equation,


(11) $$\begin{array}{l} \frac{\text{d}tr_{\text{pre}}}{\text{d}t}=-\frac{tr_{\text{pre}}}{\tau_{\text{d}}}+s_{\text{pre}}\left(t\right) \end{array}$$

where *s*_pre_(*t*) is the presynaptic spike train and *tr*_pre_(0) = 0. This can equivalently be expressed as a convolution between the exponentially decaying kernel and the presynaptic spike train


(12) $$\begin{array}{l} tr_{\text{pre}}\left(t\right)=K_{\text{d}}*s_{\text{pre}}\left(t\right). \end{array}$$

Analogous equations hold for the postsynaptic trace variable.

The trace equation (Equation 11) and its postsynaptic counterpart can be expressed practically one-to-one in NESTML syntax (Listing 3, lines 8 and 9, and 20 and 25), as can the weight update rule (Equation 9) on lines 19 and 24.

Further modifications and developments of the model have now become very easy thanks to the use of NESTML. For example, adding a dependence on the existing weight in the update rule (Equation 9) is now trivial, by inserting these new terms into the weight update expression (Listing 3, lines 19 and 24).

#### 3.1.3 Code generation

As discussed in Section 2.3, NESTML transforms the code for neuron and synapse models in such a way as to prevent redundancy of state variables that are defined in the synapse but that depends only on the postsynaptic spiking activity. To invoke the co-generation transformer in the PyNESTML toolchain, the synapse and postsynaptic neuron pair are given as code generator options (Listing 4, lines 2–4). In order to maintain maximum flexibility during network instantiation, there is no specific NESTML language syntax keyword to mark a spiking input port as pre- or postsynaptic. Instead, this information is passed in via the code generator options; in the example (Listing 4, lines 7–9), the name of the spiking input port in the synapse that will be connected to the postsynaptic neuron (here, post_spikes) needs to be explicitly passed to the post_ports code generator option. As described in Section 2.3, the co-generation transformer then recursively identifies all state variables (in this case, tr_post on line 5 of Listing 3) and corresponding update statements (Listing 3, lines 9 and 25) that can be moved into the neuron model.

**Listing 4:**
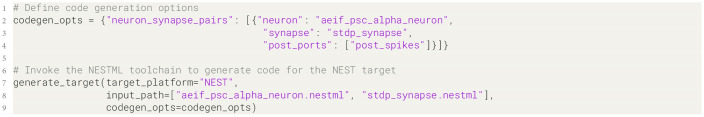
Invoking the NESTML code generator through its Python API.

#### 3.1.4 Simulation

After the PyNESTML toolchain finishes its processing, the models are available to be used in a network simulation; for example, in NEST, they can be dynamically loaded during runtime using the nest.Install() API call and can be instantiated using nest.Create() (for neurons) and nest.Connect() (for synapses).

**Figure 7:**
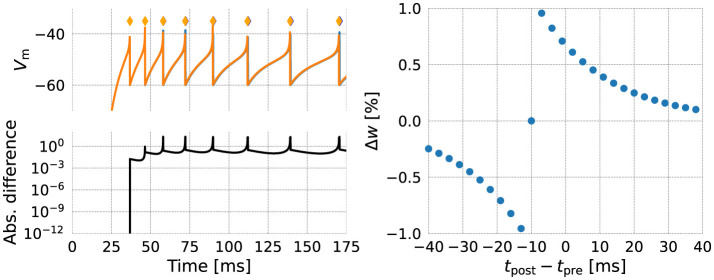
Numerical comparison of the NEST (orange) and NESTML (blue) version of each model. **(left)** Neuron model; **(right)** Synapse model.

As an initial validation step, we make a numerical comparison between the NESTML-generated code and the NEST built-in models (Figure 7, left). One neuron of each kind was instantiated, and stimulated by a step current starting at *t* = 25 ms. The membrane potential of each neuron is plotted in the top panel, and action potentials emitted after threshold crossing are indicated by diamond markers. The bottom panel shows the absolute difference between membrane potential traces, revealing differences arising primarily due to the different ways in which the firing condition is checked: the numerical solver can internally choose smaller step sizes, and in NEST, conditions are checked inside of this “inner loop”.

The NESTML generated synapse code was validated by causing pre- and postsynaptically connected neurons to fire at specific intervals (Figure 7, right). The synaptic (dendritic) delay was chosen as 10 ms, causing the horizontal offset from *t*_post_−*t*_pre_ = 0 ms. Numerical differences between the NEST and NESTML synapses were zero down to machine precision and are thus not shown.

### 3.2 Performance

#### 3.2.1 Description of the model

**Figure 8:**
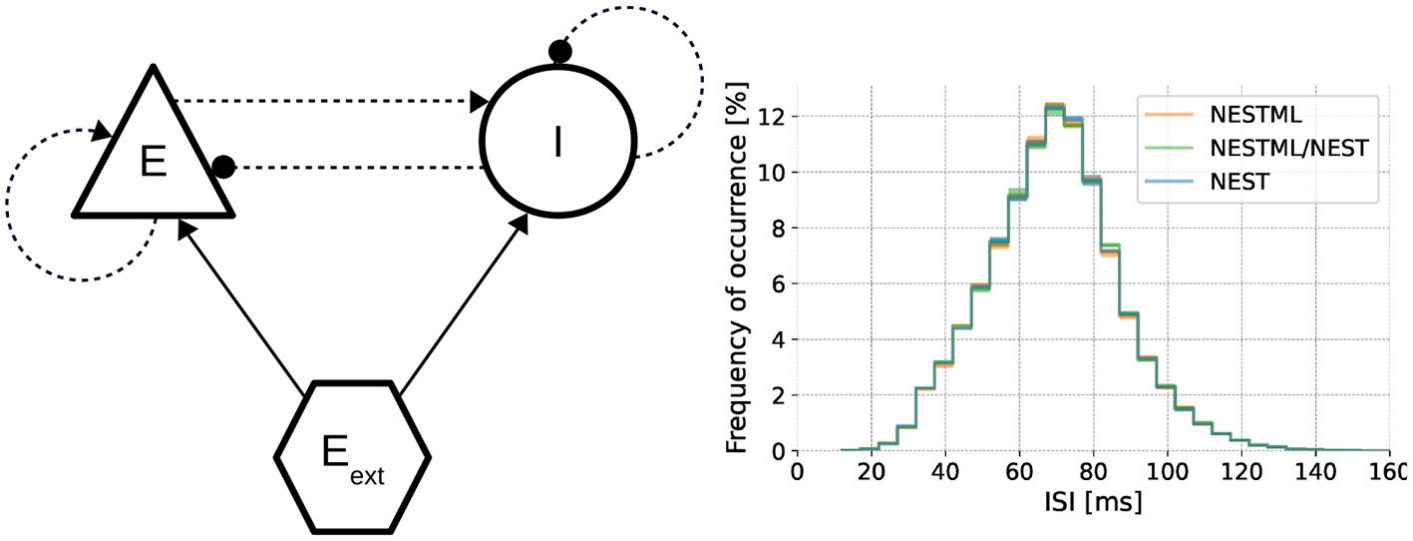
Dynamics of the neuron and synapse model in a network simulation. **Left** Architecture of the benchmark E/I network (using the graphical notation conventions from Senk et al., 2022). **Right** Interspike interval distributions for networks composed of neurons and synapses from NEST as well as NESTML validate the dynamics on a functional level (Kolmogorov-Smirnov two-sided test between NEST and the other two curves *p* < 0.01).

To benchmark how well the NESTML-generated code performs in large-scale simulations, we perform simulation runs of a balanced excitatory/inhibitory (E/I) network, as illustrated in Figure 8 (left), composed of the neuron model from Section 3.1.1 in combination with the synapse model from Section 3.1.2. Networks of varying sizes are simulated using NEST. Each network contains a random, sparse connectivity, such that each neuron in the excitatory population receives connections from 1,000 randomly picked neurons from the E/I pool, and each neuron in the inhibitory population receives connections from 250 randomly picked neurons from the E/I pool. All details and numerical values of the parameters are described in Supplementary Table 3. This is a canonical and representative use case in neuroscience, based on the seminal model proposed by Brunel (2000), and allows the network size to be varied across several orders of magnitude (with the number of synapses in the network proportional to the number of neurons), while the dynamics remain qualitatively and quantitatively constant (Figure 8, right), with an average firing rate per neuron of approximately 15 spks/s, and a coefficient of variation (CV) of approximately 0.25, close to that of a Poisson process. Although under some conditions, the network dynamics can remain stable even with plastic synapses, here we set the learning rates λ_p_ = λ_d_ = 0 for simplicity. In this way, the weight updates are computed, but the actual weight values are not modified and the resulting network dynamics remain stationary.

The networks are simulated on a high-performance computing (HPC) cluster. The simulation is distributed over multiple compute nodes that communicate via MPI. The process running on each node, in turn, performs parallel (multiprocessing) simulation based on OpenMP threads. We perform strong scaling and weak scaling experiments (Jordan et al., 2018) to assess the performance of the network with NESTML-generated models, as compared to the NEST built-in models. In strong scaling experiments, the total problem size is fixed while the number of compute nodes is varied. This gradually reduces the load on each compute node, measuring how effective extra compute hardware is in reducing the simulation time for the same network. In weak scaling experiments, the problem size per compute node is fixed. Hence, the total problem size increases proportionally to the number of compute nodes, providing a measure of how effective the simulator is in simulating ever larger networks.

For each network, we compare the following combinations of neuron and synapse models:

- NEST Simulator built-in neuron model + NEST Simulator built-in synapse model (NEST)
- NESTML neuron model + NEST Simulator built-in synapse model (NESTML/NEST)
- NESTML neuron model + NESTML synapse model (NESTML)

The NEST built-in models are based on manually written and optimized C++ code and thus serve as our reference point.

#### 3.2.2 Runtime performance

**Figure 9:**
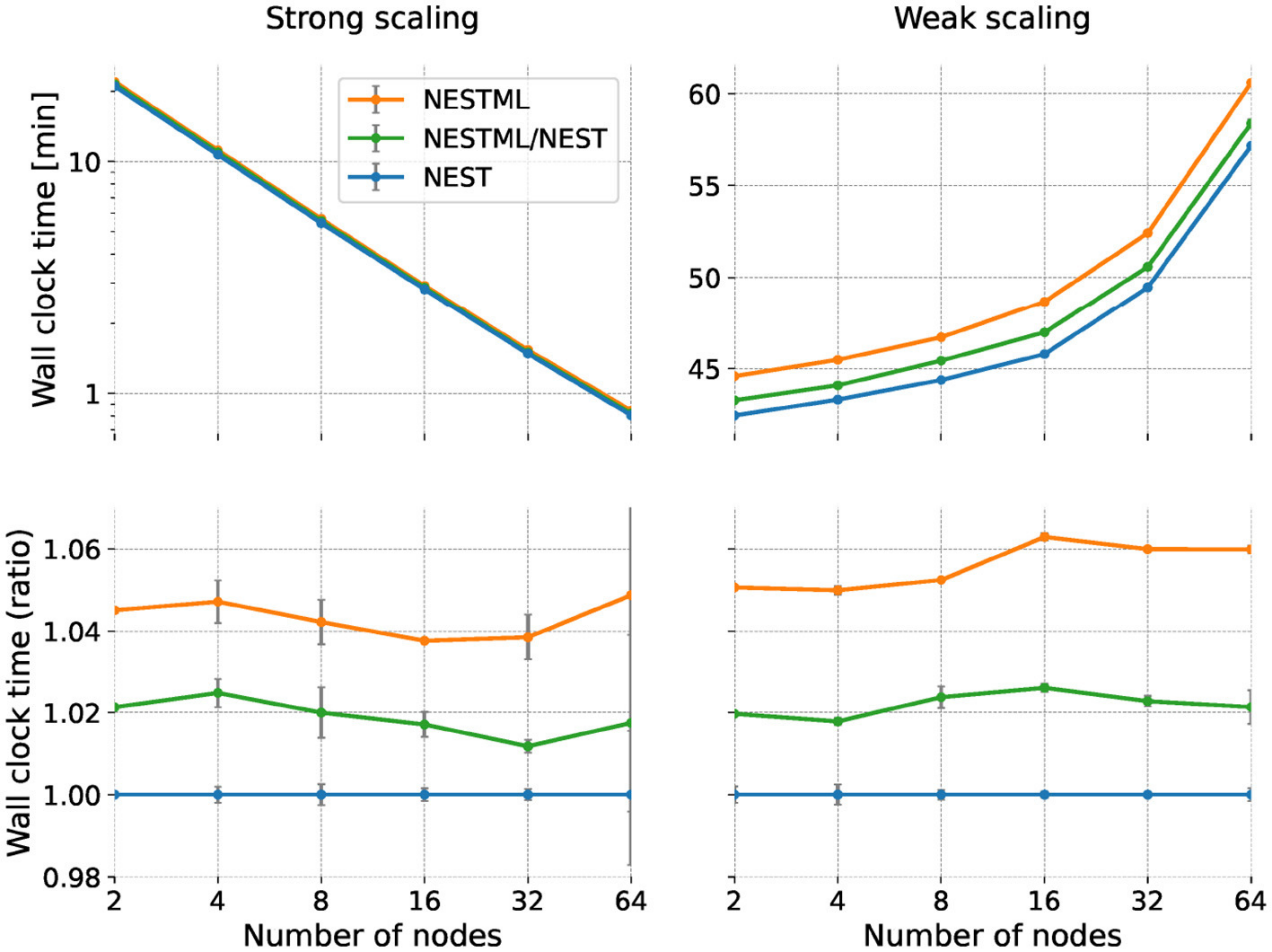
Runtime performance of the neuron and synapse models in a network simulation; comparison between NESTML-generated and NEST models. Horizontal axis indicates number of compute nodes used (same for all panels). **(Left column)** Performance in the strong scaling experiment; **(right column)** Weak scaling experiment. **(Top row)** Shows wall clock time; lower is better. **(Bottom row)** Same data but expressed as a ratio with respect to NEST.

For the strong scaling experiment, we simulate a network size of 100,000 neurons with a fixed in-degree (Figure 9, left). Since the problem size is fixed throughout the experiment, we expect the simulation to speed up when we add more compute nodes. This can be seen in the top left panel, which illustrates the wall clock time required for the simulation as a function of the number of nodes, ranging from 2 to 64. All models exhibit a near-identical reduction in wall clock time with an increasing number of nodes, demonstrating effective parallelization. Whereas on this scale, all combinations of hand-written and generated code seem to have the same performance, a small difference can be seen when plotting the ratio of the wall clock time with respect to the NEST baseline (Figure 9, lower left). Here, we can see that the NESTML/NEST code is approximately 2% slower than NEST, and the NESTML code is about 5% slower, independent of the number of nodes.

For the weak scaling experiment, we simulate the network with the network size of 100,000 neurons on each compute node with a fixed in-degree (Figure 9, right). Although the network size per compute node remains constant with the increasing number of nodes, we expect to see an increase in the total simulation time of the network due to the increasing communication and synchronization overheads that become more pronounced at larger scales. As expected, the top right panel shows the wall clock time consistently increases with the number of nodes across all configurations. Again, for ease of visual comparison, we take the NEST hand-written code as a reference and plot the same data as a ratio between the obtained wall clock time and the reference (Figure 9, bottom right). Also in the weak scaling scenario, the NEST/NESTML code is consistently around 2% slower and the NESTML code is approximately 5 to 6% slower than the NEST baseline.

As the mild performance difference between the baseline NEST code and the NEST/NESTML and NESTML variants remains approximately constant over all numbers of nodes and for both strong and weak scaling, we can conclude that it is most likely due to a slightly less efficient calculation of neuronal and synaptic dynamics rather than less efficient communication. An alternative cause of performance loss is different memory footprints, which we examine in the next section.

#### 3.2.3 Memory usage

**Figure 10:**
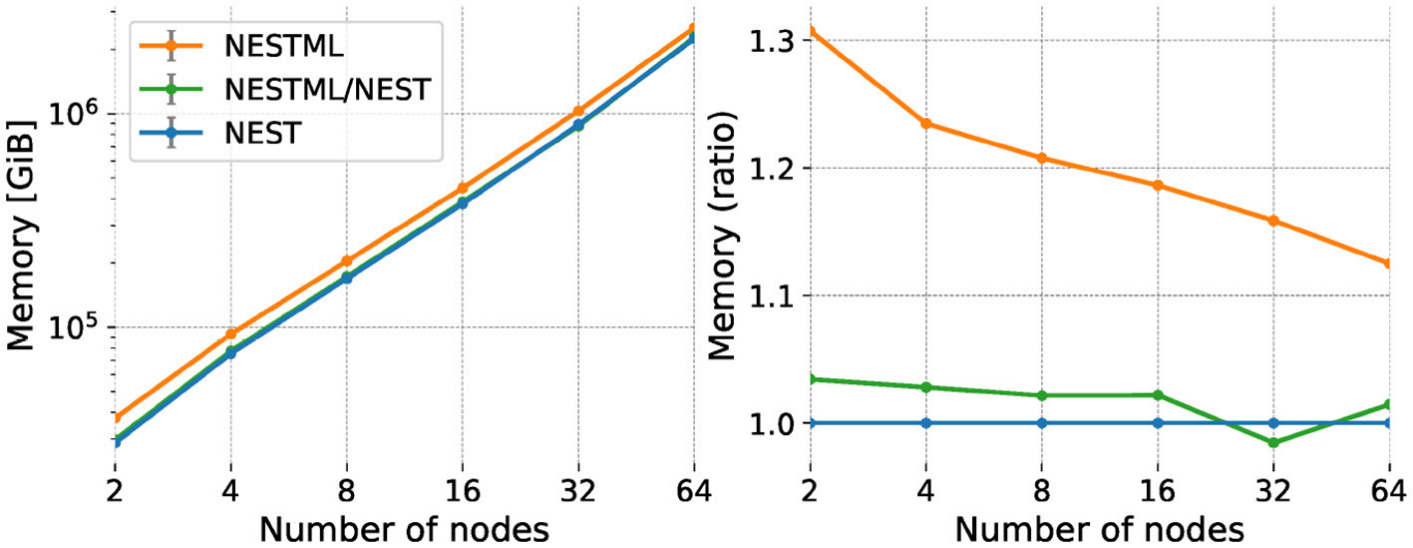
Memory use (RSS) of the network simulation for weak scaling experiment. Horizontal axis and legend are the same as in Figure 9. Vertical axis shows absolute memory usage (**left**) and memory usage expressed as a ratio with respect to NEST (**right**).

We measure the memory usage of the network during the simulation by recording the Resident Set Size (RSS) value of the simulation process. We calculate the total memory consumed during the simulation by adding the RSS values of all the processes on all the compute nodes. Memory consumption for the weak scaling experiment is shown in Figure 10, left, showing that the total value of the RSS for the simulation increases due to the increase in the overall problem size in weak scaling. The curves follow a straight line, indicating a power-law scaling relationship with a slope of approximately 1.1. As for the performance benchmarks, we plot the same data expressed as a ratio with respect to the NEST built-in models (Figure 10, right). NESTML generated code for the neurons requires up to about 5% more memory than the built-in models, while the co-generated code for neurons and synapses performs up to 30% worse, indicating that especially the synapse model code generation could benefit from further optimizations. The size-dependent effect on the memory consumption excludes a memory effect on the performance loss for NESTML reported in the previous section, as the performance loss is independent of size.

## 4 Discussion

We have created a succinct yet powerful modeling language, NESTML, and combined it with a toolchain (PyNESTML) that validates the model correctness and generates efficient, platform-specific simulation code. Separating the concerns of formulating the model and writing simulation code supports rapid development of models. The syntax of NESTML is simple and easy to learn and write, which facilitates the user in writing model code faster. It eliminates the challenges of implementing it in a native programming language (for example, in C++ for NEST), which include for instance the introduction of accidental errors due to the absence of physical units and correctness checks. The NESTML toolchain validates models, such that no physically inconsistent equations can be expressed, and contains powerful model transformers (such as the co-generation transformer) that support the generation of highly performant code. The many tests covering different aspects of the models and toolchain help correctness and decrease the chance of errors.

Both the language and the toolchain have proven themselves through real-world use cases (Schulte to Brinke et al., 2022; Hagen et al., 2022; Oberländer et al., 2022; Bouhadjar et al., 2022; Jaras Castaños, 2023), and have shown to be of practical value for research in computational neuroscience, achieving performance that is competitive with hand-written simulation code. Nordlie et al. (2009) suggest a checklist for model descriptions but do not define the desired formulation of neuron and synapse models in detail. NESTML provides exactly such a formulation, as it is expressive yet concise, easy to write, and understood by humans, yet is also precise (unambiguous) and suitable for computer simulation; properties which make it perfect for inclusion in a paper-based as well as software-based publication, something that would not be practical for an XML-based format.

Our results reveal a small performance reduction between the hand-written code of NEST and the code generated by NESTML. The hand-written NEST built-in models were developed over several years and subject to several rounds of optimization to enhance their performance. It is therefore to be expected that NESTML code, generated from generic model-agnostic templates, will miss some model-specific optimizations and therefore experience some performance loss. We believe that this slight loss is more than compensated for by the significant time savings achieved in writing and verifying the numerics of new models, as the use of NESTML allows the modeling process (Figure 1) to be carried out using the agile software engineering methodology (Alliance, 2001), emphasizing incremental development, early and continuous delivery of results, and flexibility in implementing changes. Moreover, future improvements in the toolchain could even lead to performance gains. For example, extending the set of model transformers to precompute constants needed for ODE updates could improve the performance of the generated code to be on par with, or even outperform, the hand-written NEST model code.

A standard model interchange format like NESTML helps in making models more findable, accessible, interoperable, and reusable (“FAIR” principles; Wilkinson et al., 2016). In the context of computational models, findable means that in a database of potentially hundreds of model variants, the appropriate model can be easily found. Accessible models are those that do not require extensive toolchain dependencies to work with. Interoperable models are usable across different computation hardware and simulation platforms. Reusable models are those that can be easily extended and iterated upon. These design goals are supported by the accessible, human-readable syntax of NESTML, as well as its supporting infrastructure, such as a curated model database, detailed online documentation, and continuous integration (see Section 4.1). Writing models in NESTML makes it easy for newcomers to the field to extend and adapt, rather than having to write low-level code or start from scratch. Several platforms are currently under development for the collaborative development, visualization and sharing of models, as well as a searchable database of models, for instance, Open Source Brain (OSB; Gleeson et al., 2019) and EBRAINS (EBRAINS, 2025). The wider use of NESTML as a modeling standard would facilitate interchange and promote interoperability between these software services.

Simulation software should be reliable, supporting the reproducibility of scientific results. An individual simulator should exhibit *replicable* behavior: repeated simulations of the same model should yield bitwise identical results, regardless of the number of threads or processing nodes used. In contrast, using a different numerical solver or simulating the same model on a different computer platform may alter the results, especially in network models exhibiting chaotic and unstable dynamics. However, overall, results and conclusions should be *reproducible*, obtaining the same overall quantitative and qualitative conclusions (for a commentary on this terminology, see Plesser, 2018). Recent work comparing numerical results across simulators (Gleeson et al., 2010) and examining implementation issues that are inherent to network modeling (Henker et al., 2012; Gutzen et al., 2018; Trensch et al., 2018) point to the need for a thorough suite of benchmarks for simulator testing. NESTML facilitates replicability and reproducibility studies by having a common model interchange format and a large number of unit tests, covering most of the models in the database. When cross-validating between neuromorphic platforms, the same NESTML model can be used as a basis; any differences in simulation results must then be due to differences in the platform code itself rather than the model.

### 4.1 Software development methodology

NESTML is research software, that is, software that is used in research (Hasselbring et al., 2024) (although our software license, GNU GPL v2.0 Free Software Foundation, 1991 also allows commercial use). Overall, software can be classified in various tiers: from analysis code (one-off script; often not revised after publication), to prototype (best effort maintenance) to research software infrastructure (professional product). Adhering to software engineering best practices during development helps to achieve an infrastructure level of software quality. Practices that we have found particularly helpful are:

- The processing toolchain for NESTML is designed using standard software design patterns, such as the visitor pattern for iterating over the AST, a symbol table for resolving names and scopes, and context condition checks for model validation (Section 2.5.2; Hölldobler et al., 2021). The code is well documented (using in-code comments and docstrings) and a reference manual is automatically generated in the form of a webpage.
- Our software development process follows the agile approach (Alliance, 2001). In accordance with this, new toolchain features are typically based on case studies and requests from end users. Each feature is documented from an end user perspective.
- Unit and integration tests: A battery of unit tests is run that tests the toolchain itself. If successful, a second series of tests is run that generate and build code, and run a simulation in NEST and other target platforms, allowing numerical validation of the behavioral output of all models that are part of the NESTML distribution, such as the response of neurons to input, checks on the timing of emitted spikes, and for synapses, how the weight change is dependent on the timing of pre- and postsynaptic spikes. Many of the models are tested in ways that are specific to the behavioral repertoire of that model; developers of a new model should ensure that a corresponding set of tests is created and added as part of the toolchain code alongside the model itself. Each new feature of the toolchain also includes one or more tests for that feature.
- Continuous integration (CI): A CI workflow is triggered whenever code is contributed to the NESTML codebase. In the CI environment, all unit and integration tests are automatically run. The test results are shown on the GitHub web interface; if any of the tests fail, the code is rejected. Currently, we use GitHub Actions^4^ as our CI provider.

Users of NESTML are invited to contribute to NESTML itself, adapting or adding language elements, or performing bug fixes and feature enhancements on the PyNESTML toolchain. All contributions are reviewed by at least one NESTML developer before being merged into the git (Torvalds, 2022) development branch of NESTML^5^. On a regular cycle, new NESTML software releases are published, identified by a major, minor and patch-level semantic version number (Preston-Werner, 2013) and an entry on Zenodo with DOI ^6^. Backwards-incompatible changes, such as changes to the NESTML grammar, are infrequent. In this case, the NESTML major version number is incremented (Preston-Werner, 2013), and we provide detailed instructions for how models should be adapted. Typically, a semantically equivalent form can be readily found in the knowledge graph.

In addition to the PyNESTML toolchain code and documentation, we curate a database of models, the entries of which can serve as templates or examples for further development and customization. These are distributed alongside the code in the NESTML GitHub repository^7^ and are bundled into software releases (alongside the toolchain itself). Users are invited to contribute the models they developed in their research into our central database, complemented by unit tests, documentation and usage tutorials.

### 4.2 Limitations

When new use cases are considered for implementation in NESTML, there are three categories of feasibility. The easiest class of modeling challenges is where a particular model has not yet been created in the NESTML model catalog, but implementing the model is feasible within the span of no more than a few hours.

A second class of challenges is when a model cannot be implemented in NESTML right now, but that could be made possible by adjusting or augmenting the templates, and which could be completed in a time span of weeks or months, possibly involving discussions with the NESTML development team. These challenges fit within the scope of NESTML, but the necessary features are not yet implemented. An example of such a challenge is to add support for a new simulation engine to the toolchain, or for a new plasticity rule that requires, for instance, additional buffering of state variables at previous timepoints.

Then there is a third category of maximally difficult problems, which could be possible in principle to implement, but which fall outside the NESTML scope. For example, neuron models that require an awareness of the totality of connections coming into the cell are at present difficult to implement because NESTML is not aware of the instances and connectivity of the models it defines. This makes mechanisms such as synaptic normalization, in which the norm of the vector of all synaptic weights of a cell is held constant, fundamentally unsuitable for expression in NESTML.

### 4.3 Future work

Developing and adopting standards is hard: even in simple descriptions, there are many edge cases to consider, while the standard should be flexible and generic enough to allow widespread adoption; flexibility may be key to making standards work in practice (Holmes et al., 2010). NESTML will continue to evolve, in particular in terms of its language features and support for simulation engines. Progress is underway to support the neuromorphic platform SpiNNaker-2 (Mayr et al., 2019) and to support the ability for NEST to run on GPU accelerators (Golosio et al., 2021, 2023), as well as other hardware that minimizes energy use by means of spike-based learning paradigms that are inherently robust to noise, such as those based on surrogate gradient methods (Yang and Chen, 2025).

The same mathematical model can be implemented in a number of ways in NESTML. There should be a set of design guidelines (see for instance Wimalaratne et al., 2009), complementary to our existing curated model database. In the future, even more rigorous formalization of the denotational and operational semantics of each language element in a formal calculus would allow formal, logically sound proofs about the model properties, further bolstering the FAIR principles. In general, we strive to continuously improve our documentation in terms of extent and precision.

In the broader computational neuroscience software ecosystem, we aim to integrate better with other standards, such as NeuroML (Sinha et al., 2024), by providing “source-to-source” translators or “transpilers”. For the NESTML to NeuroML translation, this can use the existing code generation facilities in NESTML.

At present, NESTML tutorials are frequently given using Jupyter Notebooks running on “the cloud”: high-performing computing resources, accessible via the internet, meaning no installation of software is necessary for the students. A full integration of NESTML into NEST Desktop (Spreizer et al., 2021), a graphical user interface (GUI) for NEST Simulator aimed at teaching, is currently underway. Using a GUI in combination with NESTML would lead to an even lower threshold for use, especially in the context of students using NESTML in the classroom as an educational tool.

## Data Availability

Publicly available datasets were analyzed in this study. This data can be found here: https://github.com/nest/nestml.

## Footnotes

^1^ https://www.w3.org/XML/

^2^ https://yaml.org/

^3^ https://nestml.readthedocs.io/en/latest/models_library/

^4^GitHub Actions: https://github.com/features/actions.

^5^ https://github.com/nest/nestml

^6^Zenodo: Research Shared; https://www.zenodo.org.

^7^NESTML GitHub repository: https://github.com/nest/nestml.
