# Supplementary material for: NESTML: a generic modeling language and code generation tool for the simulation of spiking neural networks with advanced plasticity rules
Source: Front Neuroinform. 2025 Jun 4;19:1544143. doi: 10.3389/fninf.2025.1544143 (PMC12174165; doi:10.3389/fninf.2025.1544143)
Supplement: Supplementary file 1 [file Data_Sheet_1.pdf]

Supplementary Material

1 SUPPLEMENTARY MATERIAL

1.1 Supplementary Material A: NESTML language elements

Keyword(s)	Description
integer, real, string, boolean, mV, J/(mol*K), C*J*s**-1, ...	types
min, max, exp, sinh, erfc, ...	mathematical functions
random_normal, random_uniform	draw sample from statistical distribution
if..elif..else	conditional statements
while..do, for	loop statements
function, return	definition of functions
+, -, *, **, %, |, &, <<, +=, <<=, ...	expressions, bit operators, assignment operators
', inline	ODE and inline expression definition
<, <=, !=, ...	conditional expressions
true, false, inf, "..."	literal values
[,]	vector operations
timestep, steps, t	simulation timing functions and time variable
integrate_odes	integrate ODEs
print, println	printing/logging functions for debugging
emit_spike	emit spike event
equations, input, output, update, state, parameters, internals, onReceive, onCondition,	model blocks
spike, continuous	input/output port designations

Table S1. An overview of syntactical elements of the NESTML language

1.2 Supplementary Material B: NESTML model checks

**Description**
Each called function is defined and parameter types are consistent
Each block is defined at most once
Each variable is defined at most once within each scope.
Each variable used in an expression has been defined.
All state variables are assigned an initial value
Input ports are not assigned to.
All ODEs are of order greater than 0.
Physical unit literals are correct (e.g. “1/mV” but not “2/mV”).
For files containing multiple models, model names are unique.
Spiking input ports have unique qualifier keywords; ”continuous” type input ports have no qualifiers.
All defined kernels have the type `real`.
Parameters and internals are not assigned outside the parameters (resp. internals) block.
If the `emit_spike()` function is called anywhere in the model, a spiking output port is defined.
All expressions and ODEs have consistent units on the left- and right-hand sides of the equation.
If ODEs are defined, the `integrate_odes()` function is called at least once in the model.
Functions defined in the model have consistent units and contain a ”return” statement.
The variable on the left-hand side of an ODE definition has been declared in the ”state” block.
Arguments to the convolve() function are correct.
Kernels are used only in convolutions. Dirac delta functions may only have the time t as a single argument.
All invariants evaluate to a boolean type.
Operations on vectors are consistent in terms of operating on vectors of the same length.
All declared vectors have a size greater than 0.
For synapse models, the “priority” parameter on event handlers has correct (unique and non-negative) priority values.

Table S2. List of context condition checks for NESTML models

1.3 Supplementary Material C: Alternative version of the neuron model

```
1  model aeif_psc_alpha_neuron:
2    state:
3      V_m mV = E_L                      # Membrane potential
4      I_adap pA = 0 pA                  # Spike-adaptation current
5      I_syn pA = 0 nS                   # AHP conductance
6      I_syn' pA/ms = 0 nS/ms            # AHP conductance
7
8    equations:
9      I_syn'' = -2 * I_syn' / tau_syn - I_syn / tau_syn**2
10     inline I_spike pA = g_L * Delta_T * (V_m - V_th) / Delta_T
11     inline I_leak pA = -g_L * (V_m - E_L)
12     V_m' = (I_leak + I_spike + I_syn - I_adap + I_stim) / C_m
13     I_adap' = (a * (V_m - E_L) - I_adap) / tau_adap
14
15   parameters:
16     C_m pF = 281 pF              # Membrane capacitance
17     g_L nS = 30 nS               # Leak conductance
18     E_L mV = -70.6 mV            # Leak reversal potential
19     a nS = 4 nS                  # Subthreshold adaptation
20     b pA = 80.5 pA               # Spike-triggered adaptation
21     Delta_T mV = 2 mV            # Slope factor
22     tau_adap ms = 144 ms         # Adaptation time constant
23     V_th mV = -50.4 mV           # Spike initiation threshold
24     V_peak mV = 0 mV             # Spike detection threshold
25     tau_syn ms = 0.5 ms          # Synaptic time constant
26
27   input:
28     spikes <- spike
29     I_stim pA <- continuous
30
31   output:
32     spike
33
34   update:
35     integrate_odes()
36
37   onCondition(V_m >= V_peak):     # Threshold crossing detection
38     V_m = E_L     # Reset potential
39     I_adap += b
40     emit_spike()
41
42   onReceive(spikes):              # Spike input
43     I_syn' += spikes * (e / tau_syn)
```

Listing 1: An alternative version of the adaptive exponential integrate-and-fire neuron model with alpha-shaped postsynaptic currents. In this version, spikes are processed in an event handler (lines 42-43) instead of using convolutions (compare Listing 2, lines 7 and 10).

1.4 Supplementary Material D: Balanced network model description

A: Model summary	
**Populations**	Three: excitatory, inhibitory, external input
**Topology**	—
**Connectivity**	Random convergent connections
**Neuron model**	See Section 3.1.1
**Channel models**	—
**Synapse model**	See Section 3.1.2
**Plasticity**	—
**Input**	Independent fixed-rate Poisson spike trains to all neurons
**Measurements**	Spike activity

B: Populations		
**Name**	**Elements**	**Size**
E	aeif_psc_alpha	$N_{\mathrm{E}} = 4N_{\mathrm{I}}$
I	aeif_psc_alpha	N_{I}
$\mathrm{E}_{\mathrm{ext}}$	Poisson generator	$C_{\mathrm{E}}(N_{\mathrm{E}} + N_{\mathrm{I}})$

C: Connectivity			
**Name**	**Source**	**Target**	**Pattern**
EE	E	E	Random with fixed in-degree C_{E}, weight J, delay d
IE	I	E	Random with fixed in-degree C_{E}, weight J, delay d
EI	E	I	Random with fixed in-degree C_{I}, weight $-gJ$, delay d
II	I	I	Random with fixed in-degree C_{I}, weight $-gJ$, delay d
Ext	$\mathrm{E}_{\mathrm{ext}}$	E and I	Fixed in-degree C_{E}, weight J, delay d

D: Neuron and synapse model	
**Name**	aeif_psc_alpha
**Type**	Adaptive exponential integrate-and-fire with alpha-shape postsynaptic current kernel
**Subthreshold dynamics**	See Section 3.1.1
**Spiking**	See Section 3.1.2

Neuron model parameters			
**Name**	**Description**	**Value**	**Unit**
C_m	Membrane capacitance	250	pF
g_L	Leak conductance	12.5	nS
E_L	Leak reversal potential	0	mV
a	Subthreshold adaptation	4	nS
b	Spike-triggered adaptation	80.5	pA
Δ_T	Slope factor	2	mV
τ_w	Adaptation time constant	144	ms
V_th	Spike initiation threshold	20	mV
V_peak	Spike detection threshold	20	mV
τ_syn	Synaptic time constant	0.5	ms

Synapse model parameters			
**Name**	**Description**	**Value**	**Unit**
d	Synaptic delay	1	ms
τ_tr	pre/post trace time constant	20	ms
λ_p	Potentiation learning rate	0	–
λ_d	Depression learning rate	0	–

E: Input	
**Type**	**Description**
Poisson generators	Fixed rate ν_ext, C_E generators per neuron

F: Measurements
Spike activity

Table S3: Tabular description of the balanced network in Section 3.2.1.
